# Transcriptomic Response to Water Deficit Reveals a Crucial Role of Phosphate Acquisition in a Drought-Tolerant Common Bean Landrace

**DOI:** 10.3390/plants9040445

**Published:** 2020-04-02

**Authors:** Cristina María López, Manuel Pineda, Josefa M Alamillo

**Affiliations:** Departamento de Botánica, Ecología y Fisiología Vegetal, Grupo de Fisiología Molecular y Biotecnología de Plantas, Campus de Excelencia Internacional Agroalimentario, CEIA3, Campus de Rabanales, Edif. Severo Ochoa, Universidad de Córdoba, 1407 Córdoba, Spain; b22lovac@uco.es (C.M.L.); bb1piprm@uco.es (M.P.)

**Keywords:** ABA-response, drought, legumes, *Phaseolus vulgaris*, phosphate-starvation, RNA-seq

## Abstract

Drought is one of the most critical factors limiting legume crop productivity. Understanding the molecular mechanisms of drought tolerance in the common bean is required to improve the yields of this important crop under adverse conditions. In this work, RNA-seq analysis was performed to compare the transcriptome profiles of drought-stressed and well-irrigated plants of a previously characterized drought-tolerant common bean landrace. The analysis revealed responses related with the abscisic acid signaling, including downregulation of a phosphatase 2C (PP2C) and an abscisic acid-8′ hydroxylase, and upregulation of several key transcription factors and genes involved in cell wall remodeling, synthesis of osmoprotectants, protection of photosynthetic apparatus, and downregulation of genes involved in cell expansion. The results also highlighted a significant proportion of differentially expressed genes related to phosphate starvation response. In addition, the moderate detrimental effects of drought in the biomass of these tolerant plants were abolished by the addition of phosphate, thus indicating that, besides the ABA-mediated response, acquisition of phosphate could be crucial for the drought tolerance of this common bean genotype. These results provided information about the mechanisms involved in drought response of common bean response that could be useful for enhancing the drought tolerance of this important crop legume.

## 1. Introduction

Common bean (*Phaseolus vulgaris*) is the most important legume for human consumption worldwide. It is grown throughout the world, especially in developing countries, with a large economic and social impact [1,2,3]. Bean cultivation can be done in the absence of nitrogen fertilizers under conditions of symbiotic nitrogen fixation, thus reducing the economic and environmental impact of fertilization. However, nitrogen fixation in common beans is not usually very efficient [4], mainly because symbiosis with N_2_-fixing rhizobia is particularly sensitive to adverse conditions, especially to water scarcity [5,6,7]. It is estimated that between 60 and 73% of this crop is grown in areas that suffer from drought conditions [8], and this problem is expected to worsen due to climate change [9]. In common beans, drought inhibits nitrogen fixation even earlier than photosynthesis. Besides limiting fixed nitrogen supply, drought also affects the absorption of mineral nutrients and translocation of assimilates, resulting in a drastic reduction in yield [2,10,11]. Nevertheless, due to their high dissemination and diversity, common beans exhibit enormous genotypic variability in their drought tolerance [2,8,12]. Drought tolerance has been evaluated in many bean germplasm collections, revealing the complexity of the trait, which has additive and quantitative effects, and very significant interaction with the environment [13,14], thus limiting the obtention of highly drought-tolerant cultivars.

Common bean belongs to the so-called ureidic legumes, which incorporate the nitrogen fixed in the nodules for the *de novo* synthesis of purine nucleotides [15], which are oxidized to produce the ureides, allantoin, and allantoate [16]. In these legumes, ureides are the main nitrogen transport and storage molecules [17,18]. Ureide production also increases under stress conditions as a result of the degradation of nucleotides. Recently, there have been several studies showing that ureide production could be beneficial for plants subjected to adverse conditions [19,20,21,22]. However, the accumulation of ureides has been considered for a long time as a symptom of the drought sensitivity of ureidic legumes since these compounds increase particularly in the most sensitive varieties of soybean and common bean [6,19,23]. In our group, the capacity to tolerate drought stress of four common bean genotypes was compared at the physiological and biochemical levels. We found that drought stress caused an increase in ureides in the sensitive common bean varieties but not in the tolerant ones [24]. In addition, among the compared genotypes, landrace PHA-683 behaved like a very tolerant one, with only mild symptoms of water deficit appearing after two weeks of water withholding. This genotype was able to maintain unaltered N_2_ fixation after 7 days without irrigation, and the nodule activity was only partially inhibited after two weeks of stress. Moreover, these plants did not accumulate ureides, even after two weeks of stress [24].

Most efforts to obtain drought-tolerant legumes have been made using a phenotypic or genotypic characterization, based on the analysis of a discrete number of parameters. However, molecular analyses are required to understand how drought tolerance is achieved in ureidic legumes. In recent years, transcriptome sequencing has emerged as a powerful tool for providing high-resolution data and transcription networks widely applied in the analysis to developmental or environmental responses in many crops, including some legumes [25,26,27], but only a few transcriptome analyses have considered the tolerance to drought in common bean [28,29,30].

The hypothesis of this work was that investigating the molecular mechanisms of drought tolerance in common bean genotypes known to exert high tolerance levels would help to discover key factors that could be used in the amelioration of abiotic stress effects in this important crop.

We used the common bean PHA-0683 landrace, recently shown to maintain active nitrogen fixation and to retain high relative water content in their tissues, until severe water stress was imposed [24]. To decipher the molecular changes associated with drought tolerance, the genome-wide expression analysis using RNA-seq in response to drought in PHA-0683 plants was done. The analysis of the differentially expressed genes (DEGs) and the functional gene ontology (GO) enrichment between control and drought-stressed plants revealed a prevalent relevance of genes related to phosphate nutrition stress in response to water deficit of this tolerant genotype.

## 2. Results

To elucidate the molecular strategies displayed by common bean landrace PHA-683 to tolerate drought conditions, the RNA-seq approach was chosen to compare the genome-wide changes in transcript levels in response to water deficit. Since, in our previous work, these plants did not show any apparent drought symptoms after 7 days of water stress [24], 10 days of water deprivation was chosen as the optimal stress length to investigate the changes in gene expression associated with early events of water deficit in these plants. Plants cultured under N_2_ fixation conditions were regularly irrigated until they were 28 days old, and then they were randomly distributed into two groups, one kept under regular watering, whereas the other one received no further irrigation for 10 days. Total RNA from three independent biological replicates from each treatment was used to obtain the mRNA fraction from control and 10-days-drought-stressed trifoliate leaves. The mRNA was then copied and amplified into six independent cDNA libraries. The transcriptome changes of control and drought-stressed leaves were examined using the Ion-Torrent RNA-Seq technology. The total number of raw, pre-processed reads ranged from 32 to 37 million reads per library, with an average length of 135 bp. After the removal of low-quality reads and adapter sequences, clean reads were aligned with the *P. vulgaris* L. v2.1 reference genome obtained from the *Phytozome* website (http://www.phytozome.net/) [31]. The expression levels of the genes in leaf samples from 10 days-drought-stressed were compared to control well-irrigated plants, and a total of 211 differentially expressed genes (DEGs) were found, with a Log2FolChange > 1, and a *p*-value adjusted to multiple testing < 0.01 (Supplementary Table S1).

### 2.1. Functional Annotation and Gene Ontology Enrichment of DEGs upon Drought Stress

**Figure 1:**
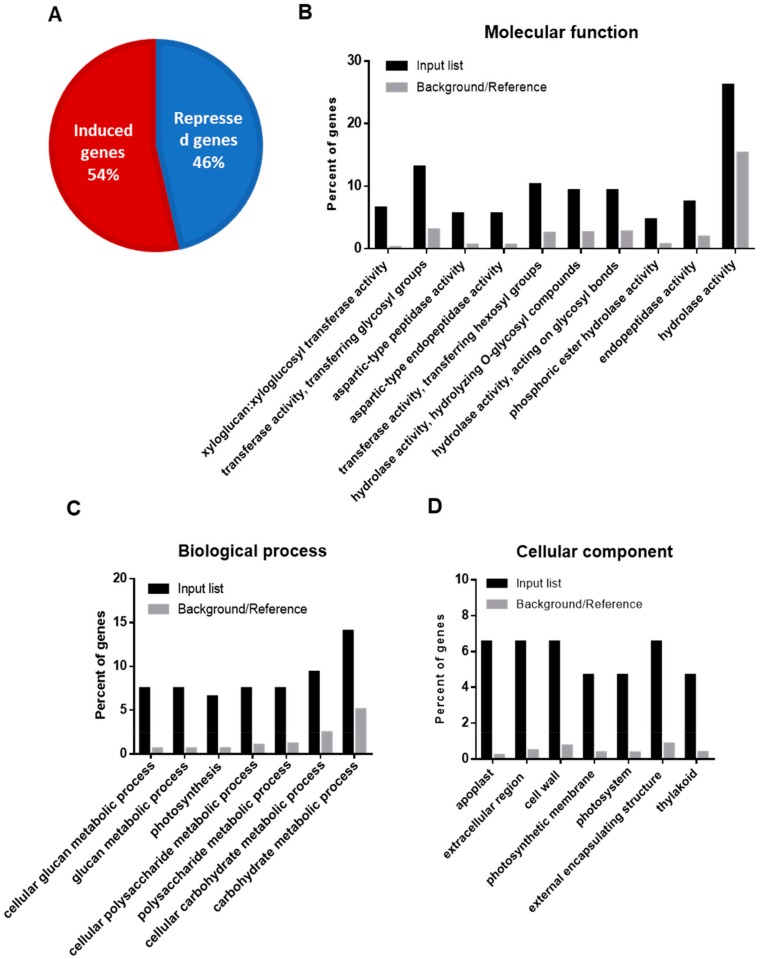
(**A**) Diagram showing percentage (%) of induced (red) and repressed genes (blue) in *P. vulgaris* leaves in response to drought. (**B**) Molecular functions, (**C**) biological process, and (**D**) cellular component gene ontology (GO) enrichment categorization of the differentially expressed genes in RNA-seq analysis using the AgriGOv2.0 software (http://bioinfo.cau.edu.cn/agriGO/) [32].

Among the annotated genes, showing significant differential expression in drought and control samples, 47% were found upregulated, and 53% of DEGs appeared downregulated (Figure 1A). To find out a biological significance of DEGs during drought, we made a gene ontology (GO) enrichment analysis of up- and downregulated genes in relation to molecular function, biological process, or cellular component using AgriGO v2.0 [32] (Figure 1B–D). Among the molecular function enriched terms, the most prevailing ones were those related to hydrolase activity, with phosphatase, followed by glycosyltransferase and endopeptidase activities (Figure 1B). According to the biological process, the most enriched one concerned carbohydrate or polysaccharide metabolic processes (Figure 1C). The most enriched cellular component was the extracellular region, including apoplast and cell wall components, followed by thylakoid membranes (Figure 1D).

**Figure 2:**
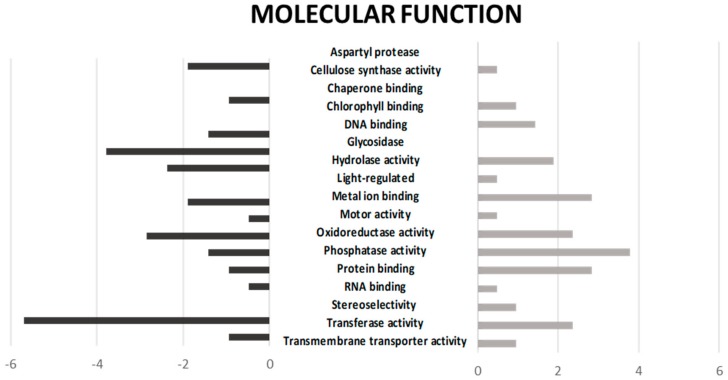
Molecular function depicted as a percentage of induced (grey bars) and repressed (black) differentially expressed genes.

To further dissect the overrepresented molecular functions that change among drought and control samples, the number of induced and repressed genes were depicted (Figure 2). Interestingly, the highest proportion of induced genes corresponded to phosphatase activity categorization, whereas repressed genes were mostly those of transferase and glycosidase activities. The closest view of the enriched molecular functions revealed that, besides phosphatases, there were also a significant number of phosphate homeostasis-related genes, including proteins involved in phosphorous nutrition, which were either induced or repressed in response to the drought treatment according to the RNA-seq results (Supplementary Table S1), revealing that the regulation of phosphate homeostasis was a crucial event in response to drought stress in this common bean landrace.

Interestingly, besides the phosphate-related genes, 6% of the DEGs were involved in cell wall modification, including two coding for a cellulose synthase H1—*Phvul.005G117833* and *Phvul.005G116501*—that were induced 9.8 and 3.2 fold, respectively, in the drought-treated samples and several downregulated genes coding for extensins (*Phvul.004G161500*) and for xyloglucan endotransglucosylase hydrolases belonging to expansins family (eight genes), thus indicating that drought caused important changes in the cell wall structure.

Among the induced glycosyltransferases, there was also one galactinol synthase (*Phvul.007G203400*) involved in the biosynthesis of the raffinose family oligosaccharides (RFOs) that function as osmoprotectants [33].

On the other hand, 4% of the DEGs was found to belong to transcription factors (TFs), including the induction of a WRKY (*Phvul.007G046800*) and a MYB *(Phvul.003G028000)* and the downregulation of six putative TFs belonging to the MYB, NAC, and LHDH families. Moreover, 6% of DEGs were found to correspond to kinases, phosphatases, calcium-binding and protein receptors that could be involved in the early signaling of the stress responses. Among these, there was a downregulation of a putative phosphatase 2C (*Phvul.001G021200*). Downregulation was also found for a putative abscisic acid 8′-hydroxylase (*Phvul.002G122200*), involved in the degradation of abcisic acid (ABA), thus suggesting the upregulation of ABA-mediated responses.

There was also a significant proportion of genes related to photosynthesis, including several light-harvesting, chlorophyll-binding proteins, which could be related to the protection of photosynthetic complexes, as well as several stress-related genes, such as glutathione S-transferase, small heat shock proteins, chaperones, and others. Finally, 12% of the DEGs encoded putative proteins of unknown functions.

### 2.2. Validation of Changes in the Expression Levels by qRT-PCR

**Figure 3:**
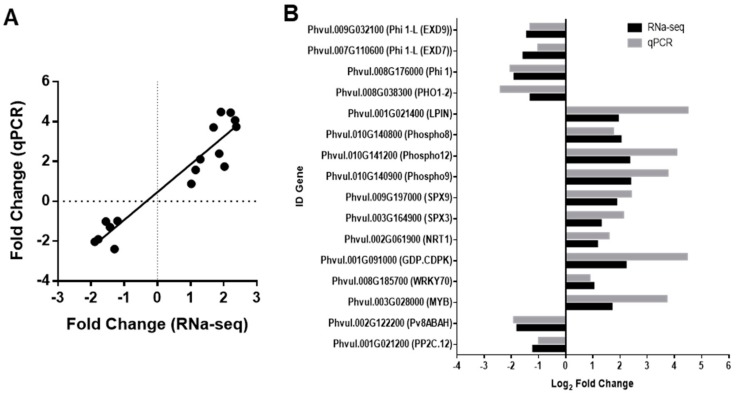
Validation by qRT-PCR of the changes in expression in response to drought of several genes identified in the RNA-seq. (**A**) Pearson correlation between RNA-seq and qRT-PCR gene expression values of selected differentially expressed genes (DEGs). (**B**) Graphical representation of overexpressed (positive fold change) and the repressed (negative fold change) genes: *Phvul.001G021200 (PvPP2C.12); Phvul.002G122200 (Pv8′ABAH); Phvul.003G028000 (PvMYB); Phvul.008G185700 (PvWRKY70); Phvul.001G091000 (PvGDP.CDPK); Phvul.002G061900 (PvNRT1); Phvul.003G164900 (PvSPX3); Phvul.009G197000 (PvSPX9); Phvul.010G140900 (PvPhospho9); Phvul.010G141200 (PvPhospho12); Phvul.010G140800 (PvPhospho8); Phvul.001G021400 (PvLPIN); Phvul.008G038300 (PvPHO1-2); Phvul.008G176000 (PvPhi 1); Phvul.011G004400 (PvPhi 1-L (EXD7)); Phvul.009G032100 (PvPhi 1-L (EXD9)).*

Real-time quantitative PCR was conducted using gene-specific primers (Supplementary Table S2) to validate the expression patterns revealed by the RNA-seq analysis. Sixteen genes were selected from the list of DEGs, and the relative expression of target genes was calculated by the 2^−∆∆CT^ method [34] as the mean ± sd from the results of three independent biological replicates. As shown in Figure 3A, results found in the RNA-seq analysis fully correlated with those found in the qPCR (R square of 0.92) for all the selected genes. Among the DEGs whose pattern of expression was validated by qRT-PCR, were the genes related to ABA responses, as the PP2C (*Phvul.001G021200)* and the putative ABA 8′-hydroxylase gene *(Phvul.002G122200)* appeared as repressed in the drought condition. The expression of the WRKY 70 (*Phvul.008G185700)* and the MYB *(Phvul.003G028000)* transcription factors upregulated upon the drought stress in the RNA-seq and was also induced in the qRT-PCR.

A relevant amount of RNA-seq DEGs appeared as related to phosphate (P) nutrition; therefore, the expression levels of several of the phosphate-related genes were included in the list of genes whose changes in expression levels were validated by qRT-PCR. Among them, there were three genes coding for pyridoxal phosphate or PDX-related protein phosphatases, belonging to the Phospho1 or PS2 inorganic pyrophosphatase 2-like gene family [35]; we named the genes as Phospho8 (*Phvul.010G140800*), Phospho9 (*Phvul.010G140900*), and Phospho12 (*Phvul.010G141200*), which were induced in the RNA-seq and also appeared as highly induced in the qRT-PCR analysis (Figure 3B). There were also two genes related to the phosphorous starvation sensing or SPX domain proteins [36], which we named SPX9 (*Phvul.009G197000*) and SPX3 (*Phvul.003G164900).* According to the qRT-PCR results, the expression level of these genes was also heavily induced in the drought-stressed samples (Figure 3B).

The induction was also found for *PvLPIN* (*Phvul.001G021400*), a putative phosphatidate phosphatase that has been related to critical responses to phosphate starvation [37], and for *PvGDP-CDPK* (*Phvul.001G091000*), a glycerophosphodiester phosphodiesterase related to a multifunctional cyclin-dependent kinase, putatively involved in maintaining cellular phosphate homeostasis under phosphate starvation [38] (Figure 3B). In contrast, downregulation was found for the phosphate transporter-related gene, *PvPHO1-2* (*Phvul.008G038300*) (Figure 3B), and several genes coding phosphate-induced genes as *PvPHI* and *PvPHI*-like belonging to the EXORDIUM like protein family [39,40], from which two of them are shown in Figure 3B *(Phvul.007G110600; Phvul.009G032100).* Moreover, there was upregulation of the dual nitrate transporter NRT1 (*PvNRT1*, *Phvul.002G061900*) (Figure 3B), which has recently been described as an integrator of nitrate and phosphate signaling networks [41].

### 2.3. i Expression changes of Phosphorous homeostasis related genes in Drought-Stressed Plants upon Phosphorous Supplementation

To investigate if phosphate starvation was the key factor for the induction of phosphate-related genes in the drought-stressed plants, an experiment was performed in which a group of plants was supplemented with a higher concentration of P in the irrigation solution during one week just before the water deficit treatment, and the effect of phosphate addition was analyzed.

**Figure 4:**
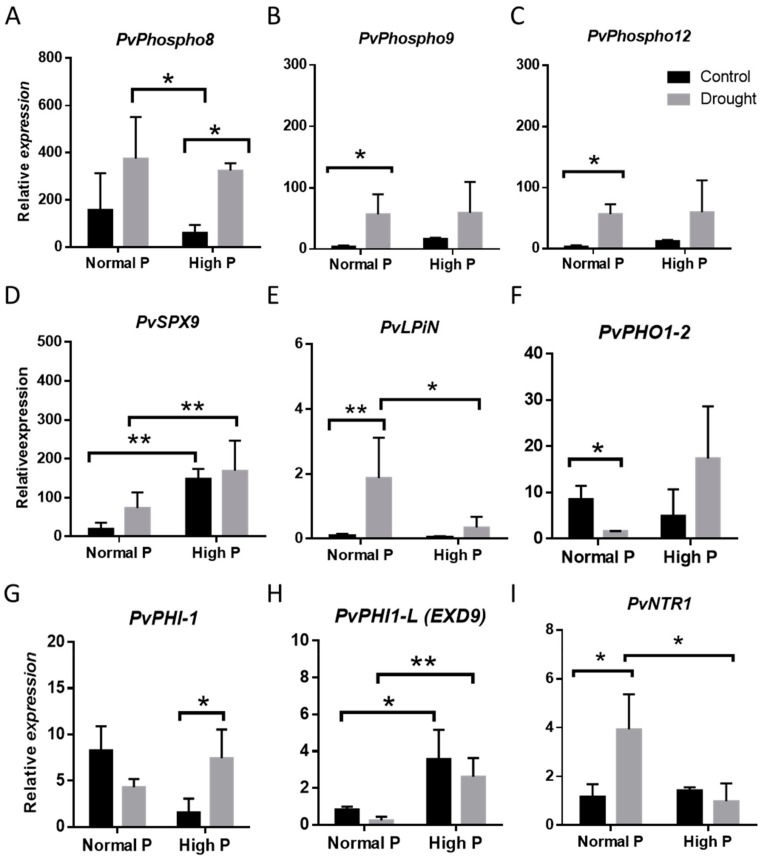
The relative expression of phosphate starvation-related stress genes in leaves from control and 10 days-drought-stressed plants that were grown under symbiotic nitrogen fixation conditions with 82 µM of KH_2_PO_4_ (Normal P) or received 200 µM of KH_2_PO_4_ (High P) for one week, just before the drought treatment. (**A**–**C**) Relative expression of *PvPhospho8, PvPhospho9, PvPhospho12*; (**D**) *PvSPX9;* (**E**) *PvLPIN;* (**F**) *PvPHO1-2;* (G) *PvPHI-1*; (**H**) *PvPHI1-L or EXORDEUM 9*; (**I**) *PvNTR1* expression in response to P supplementation and drought. Data are means of three independent experiments. Asterisks indicate statistically differences (* p < 0.05) and (** p < 0.005).

Control and water-stressed plants with and without the addition of a higher concentration of P were collected 10 days after the drought treatment, and the expression of a group of the phosphate-related genes was determined. As shown in Figure 4A–C, the expression of the three inorganic pyrophosphates (Phospho 8, 9, and 12) of the Phospho1 family was induced to similar levels in the drought-stressed samples from plants irrigated with the lower (standard P levels) and with the higher P concentration, thus suggesting that drought was more determinant than P availability for the induction of these genes.

In contrast, the supplement of phosphate prior to the drought treatment significantly attenuated the induction of the phosphatidate phosphatase (*PvLPIN*) gene (Figure 4E), suggesting that P limitation was the main condition for the upregulation of this gene. In addition, the SPX 9 and the phosphate-induced genes PHO1-2, PHI1, and PHI-L (EXD 9) (Figure 4F–H) did also show significant differences in response to drought among the normal and the high P conditions. The addition of phosphate was able to mitigate the downregulation caused by the water stress of several of the phosphate responsible (PHI and Phi-like) genes, suggesting that phosphate cellular level was the key regulatory factor for these genes. Interestingly, the expression of the dual nitrate transporter *NTR1.1* was not induced in response to drought in the high phosphate samples, indicating that phosphate level also controlled the expression of *PvNTR1.1* (Figure 4I).

**Figure 5:**
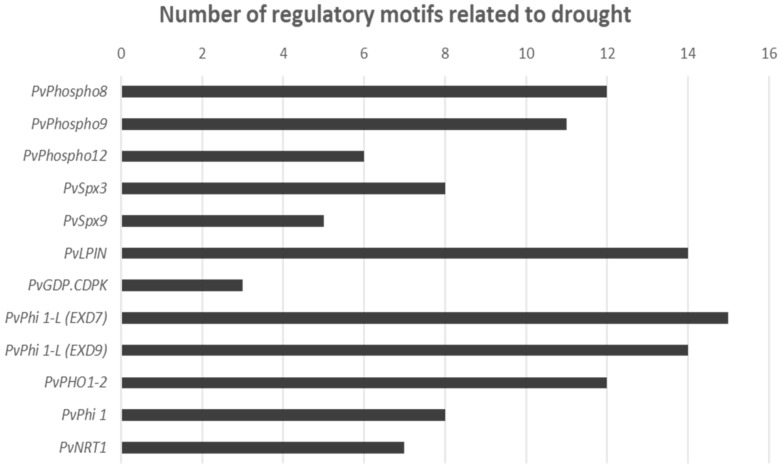
Representation of the number of regulatory motifs related to drought in the promoter sequences of the phosphate starvation related DEGs. Promoter sequences of the genes were retrieved from Phytozome (https://phytozome.jgi.doe.gov/pz/portal.html#), and 1500 bp upstream the ATG of each gene was analyzed using the PlantCARE bioinformatics platform of plant regulatory motives search (http://bioinformatics.psb.ugent.be/webtools/plantcare/html/) [42].

In order to further investigate why the expression of several phosphate-related genes was mainly regulated by water deficit, a search for water-stress *cis*-regulatory motives on the upstream genomic sequences of these genes was done using Plant Care software [42]. As shown in Figure 5, the search for regulatory motives present in the -1500 bp upstream the ATG of the promoter regions of the investigated genes revealed a significant number of drought, ABA, or osmotic stress-related motives (Supplementary Table S3), ranging from 3 motives in the promoter of the glycerophosphodiester phosphodiesterase (*PvGDP-CDPK*) up to 15 motives in the upstream sequence of LIPIN coding gene (Figure 5 and Supplementary Table S3).

### 2.4. Physiological Effects of Phosphorous Supplementation on Drought-Stressed Plants

**Figure 6:**
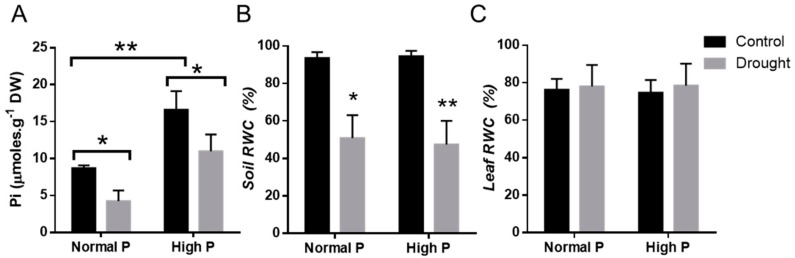
(**A**) Determination of inorganic phosphorus (Pi) concentration (µmoles.g^−1^ dry weight (DW)) in *P. vulgaris* leaves that were well-watered (control) and drought-stressed (drought) and cultivated with 80 µM of KH_2_PO_4_ (Normal P) or supplemented with 200 µM of KH_2_PO_4_ (High P) for one week before the water withholding treatment. (**B**) Soil relative water content. (**C**) Leaf relative water content measured in the 5th trifoliate leaves of control and drought-stressed plants of low or high P conditions. Data are means of three independent experiments. Asterisks indicate statistically significant differences (*p* < *0.05*).

In addition to the molecular response to drought, we also determined how much of the phosphate supplementation reached the shoots of the treated plants. The content of Pi was about twice higher in phosphate-supplemented well-irrigated plants. As expected, drought drastically reduced the amount of Pi in the leaves of the treated plants, although it was significantly higher in the drought-stressed plants that received the Pi supplement compared to the samples grown under standard P (Figure 6A). Drought reduced the Pi content to about 50% in both the low and the high P samples, although in the latter, the available Pi remained higher than the one in control irrigated samples of the low P (Figure 6A).

Relative soil water content (SWC) was reduced to near 50% in the drought-treated samples, both in the P supplemented and in the lower P pots (Figure 6B), thus demonstrating that drought condition was similar in the two groups of plants. In addition, the relative water content in leaves (RWC) was measured in the four groups of plants. As shown in Figure 6C, leaf’s RWC was maintained in the drought-stressed plants, both with and without the addition of P. This result further confirmed that, despite the low SWC, these highly tolerant plants were able to retain their RWC under these stress conditions, as previously observed for this landrace in [24].

**Figure 7:**
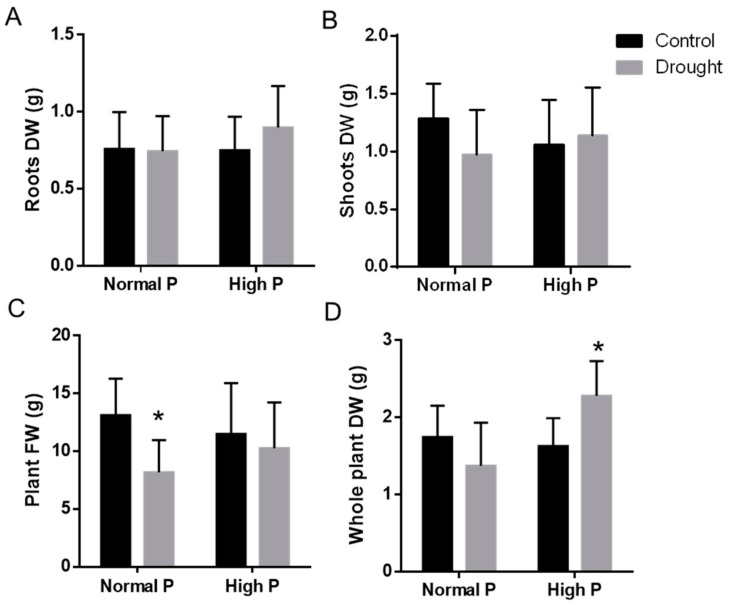
Effects of 10 days-water-deficit in the biomass of *P. vulgaris* plants that were well-watered (control) or drought-stressed (drought) cultivated with 82 µM KH_2_PO_4_ (Normal P) or irrigated with 200 µM of KH_2_PO_4_ (High P) before the drought treatment. (**A**) Biomass of roots after removal of nodules, (**B**) shoots biomass, (**C**) whole plant fresh weight, and (**D**) whole plant biomass of well-watered (control) and 10 days-drought-stressed (drought) plants. Data are means of three independent experiments with 10 plants from each treatment. Asterisks indicate statistical differences *(p < 0.05*).

To check whether increasing phosphate availability could ameliorate the moderate negative effects of drought in landrace PHA-683, plant biomass was measured in control and 10 days-drought-stressed plants with and without the addition of phosphate supplement (Figure 7).

Drought did not produce significant changes in root biomass in this landrace, although slightly higher root biomass was found in the drought-stressed high P plants compared to the control or to the normal P plants (Figure 7A). Similarly, drought caused only a slight reduction in the shoot biomass of the lower P plants, although the effect did not reach statistical significance (Figure 7B). As expected, drought reduced the fresh weight of the whole plants. However, the reduction was only significant between the drought and control plants of the lower P nutrition (Figure 7C). Moreover, the whole plant dry weight of drought-stressed high P plants was significantly higher than in the normal P stressed ones (Figure 7D). These results suggested that drought strongly affected P nutrition and that, at least in part, the moderate negative drought effects on plant biomass of this tolerant common bean landrace could be alleviated when higher P concentration is supplied.

## 3. Discussion

Functional genomic tools, such as whole-genome sequencing of transcripts, are currently one of the most useful technologies to clarify the molecular mechanisms of complex traits, such as drought tolerance and, ultimately, to obtain more efficient crops in conditions of abiotic stresses. Transcriptomic analysis, although scarce so far in legumes, has revealed new discoveries associated with the differential expression of genes not easily anticipated with previous physiological studies [25,28]. In this work, we did an RNA-seq analysis to dissect the molecular responses to water stress in a common bean landrace, previously characterized as highly tolerant [24]. The first surprising result was that, besides the large number of clean reads in each of the sequenced libraries (Supplementary Figure S1), there was only a moderate number of genes that showed differential expression compared to control plants (Figure 1A). As previously shown [24], PHA-683 landrace did not show any physiological symptoms of water stress at 7 days and only moderate symptoms at 14 days of water deprivation. Therefore, only those changes in gene expression related to early or mid-response to stress could be found after the 10 days of water deprivation used in this study. Therefore, the induced and repressed genes found in this study suggested a molecular readjustment in response to the stress in this tolerant plant.

Interestingly, among the genes that change their expression, there were downregulated genes (possibly related to ABA-mediated signaling), such as the PP2C, which is a key negative regulator of ABA signaling [43]. Similarly, there was an ABA 8′-hydroxylase gene implicated in ABA catabolism that also appeared repressed by drought (Figure 3). The downregulation of genes putatively forming part of the negative regulators of the core ABA signaling strongly suggested that this tolerant genotype could maintain an efficient ABA response to cope with water stress. Moreover, there were changes in several other possible signaling-related genes, such as genes coding for calcium-binding proteins, membrane receptors, protein kinases, and transcription factors (Supplementary Table S1), thus suggesting that they could be involved in the early or mid-responses to the stress. Among the TFs, we found significant induction of MYB and WRKY, previously related to ABA-mediated drought responses [44,45,46] and to phosphate deficiency responses [47]. Interestingly, WRKY70 has been found to be involved in both brassinosteroids-regulated plant growth and drought responses [48] and has been reported as a negative regulator of plant senescence [49]. Therefore, WRKY70 could be a key TF whose induction, together with the several DEGs related to stabilization of photosynthetic complexes and membrane and cell wall-associated changes, could be relevant for the high tolerance of this landrace.

Cell wall remodeling under drought stress is a common response in plants [50,51]. The plant cell wall is a complex structure with critical functions in plant life. The cell wall maintains the structural integrity of the cell by resisting internal hydrostatic pressures while also providing flexibility and supporting cell division and expansion. Many of the genes whose expression changed in response to water-stress in this study were related to the cell wall or extracellular proteins. Drought impacted the water potential of the cell, inducing changes in wall polymer structure and composition, thus justifying the changes in expression of genes coding several expansins, xyloglucan endotransglucosyl hydrolases, extensins, and intrinsic membrane proteins found in the RNA-seq. Due to the high complexity of cell wall and membrane interactions, analysis of these drought-mediated DEGs would require further investigations. In addition, as the cell wall is a strong sink for carbohydrates, it would be interesting to evaluate the relationships among the carbohydrate metabolism-related DEGs found in this study and the cell wall-related changes. Interestingly, several of the genes whose expression changed in the drought treatment code for PHOSPHATE-INDUCED PROTEIN1 (PHI, and PHI-Like) that form part of the large EXORDEUM-like family of genes related with brassinosteroids-mediated cell expansion [39,40]. Five of these genes were found downregulated in the RNA-seq, according to the negative effect of water stress on cell expansion (Supplementary Table S1).

In addition to the genes coding for Exordeum-like proteins that belong to the phosphate-induced protein 1 (PHI-1), there was a large proportion of the DEGs that were related to phosphorous starvation, thus supporting the relevance of phosphate acquisition for the drought tolerance of this common bean genotype. It has been reported that N_2_-fixing legumes require more P than legumes growing on mineral N, and that root nodules are strong P sinks in legumes. Thus, P concentration in the nodules of soybean [52] and white lupin [53] from P-deficient plants reach up to 3-fold that of other plant organs. Moreover, P deficiency has a strong detrimental effect on nitrogen fixation in several legumes, including common bean [54]. As shown previously [24], nitrogen fixation was only partially inhibited after a severe drought in landrace PHA-863; therefore, a large amount of P was expected to be required to maintain N_2_ fixation under these stress conditions. As drought reduced the acquisition of mineral nutrients, including P (Figure 6A), the remobilization of internal P stores, by induction of the several inorganic phosphatases, the lipid phosphatase (LPIN), and the glycerophosphodiester phosphodiesterase found in this work, might help to supply the required P to the N_2_ fixing nodules, thus contributing to the tolerance of this genotype. Accordingly, the upregulation by the drought of the expression of the LPIN and phosphodiesterase was abolished in plants growing with a higher amount of P (Figure 4).

However, the supplement of higher P concentration did not reduce the drought-mediated induction of the three phosphoethanolamine/phosphocholine phosphatase/*Phospho1* (*PvPhospho*) inorganic pyrophosphatase 1-related genes, indicating that drought was the main factor regulating the expression of these genes. The PHOSPHO1 protein belongs to pyridoxal phosphate PDX family involved in the synthesis of Vitamin B_6_ (pyridoxine and its vitamers) that has been implicated in the defense against cellular oxidative stress caused by abiotic stresses, such as drought, chilling, high light, and ozone [35], and plants with an enhanced level of vitamin B_6_ have an increased tolerance to oxidative stress and increased resistance to paraquat and photoinhibition [55]. Although further experiments will be required to determine whether the tolerant plants accumulate vitamin B_6_ in response to stress, induction of the *Phospho1* or *PDX* genes found in this study suggested their implication in the protection of the photosynthetic systems under drought stress through the synthesis of vitamin B_6_, as well as helping to supply P through their phosphatase activity. Moreover, induction of pyridoxal phosphate or other vitamin B_6_-related compounds, besides acting as a cofactor for many enzymes, is also involved in the synthesis of choline, a precursor in the synthesis of important osmolytes, such as glycine betaine. Interestingly, increasing glycine betaine accumulation has been shown to modulate the phosphate homeostasis in tomato plants [56].

The effect of phosphate addition was apparent in the group of phosphate-induced genes, *PvPHI-1* and *PvPHI1*-like of the EXORDEUM-like protein family, whose expression levels were reduced under drought in the lower P conditions but not in the P-supplemented ones (Figure 4). As previously mentioned, EXORDEUM proteins are involved in brassinosteroid-mediated cell expansion [40]. Interestingly, the supplement of phosphate was shown to alleviate the slight negative effect of drought in the biomass of this tolerant plant (Figure 7). Therefore, it is tentative to speculate that the higher expression level of the EXORDEUM-like coding genes could be related to the higher growth of the P-supplemented plants, even under drought conditions. Nevertheless, although we still do not have a mechanistic explanation on the actual role of regulation of phosphate homeostasis in the tolerance to drought, our results indicated that increasing phosphate availability reduced the negative effect of drought in the biomass of this tolerant plants (Figure 7), thus suggesting that phosphate limitation was among the main constraints caused by drought for the growth of these plants. The regulation of the phosphate nutrition-related genes in response to water deficit was further supported by the presence of several *cis*-regulatory motives found in their promoter sequences (Figure 5). It would be interesting to study whether the phosphate nutrition-related genes found in this study are also induced in plants fed with nitrate, lacking the strong phosphate sink of the nodules. Similarly, future experiments should be done comparing the induction of these genes in the tolerant and drought-sensitive plants, to ascertain whether the induction of genes involved in the mobilization of phosphorous from cell stores is a factor contributing to the drought tolerance of this genotype. Accordingly, there are reports indicating that selection for drought resistance in common bean also improves yield in phosphorus limited environments [8]. Interestingly, P supply has been previously shown to improve legume performances against soil environmental stress factors [57].

The accumulation of ureides has been considered for years as a symptom of the drought sensitivity of ureidic legumes since these compounds increase particularly in the most sensitive varieties of soybean and common bean [23,24]. Synthesis of ureides takes place in the nodules from the oxidation of the de novo synthesized purine nucleotides that incorporate the fixed nitrogen [15,16]. However, in the sensitive common bean plants, drought inhibits nitrogen fixation, and degradation of stored purine nucleotides is the source of the accumulation of ureides [18,19,24]. However, drought-stressed tolerant plants of landrace PHA-683 do not accumulate ureides and maintain N_2_ fixation under these conditions [24]. Accordingly, we did not find any changes in the expression of genes related to ureide synthesis or in the metabolism of purine nucleotides. Interestingly, there was only a reduced number of DEGs related to macromolecules degrading enzymes, such as peptidases, but a total absence of nucleases in the drought-stressed leaves, agreeing to the lack of ureides accumulation in response to stress and the highly tolerant behavior of this landrace.

In summary, RNA-seq analysis of the drought-tolerant landrace PHA-683 in response to drought revealed responses related with the ABA signaling, including upregulation of several key TF, remodeling of cell walls, synthesis of osmoprotectant oligosaccharides, protection of photosynthetic apparatus, and downregulation of genes involved in cell expansion, but, above all, there was a significant proportion of DEGs related to phosphate starvation response, thus suggesting that acquisition of phosphate could be crucial for the drought tolerance of this common bean landrace. In conclusion, the molecular analysis on a drought-tolerant common bean genotype presented here revealed the importance of phosphorous homeostasis, as well as several other key factors, in response to water stress. These results might be used in the future search for drought-tolerant genotypes or in breeding programs with an aim to obtain highly tolerant common bean plants.

## 4. Materials and Methods

### 4.1. Plant Material and Growth Conditions

In this study, a previously characterized drought-tolerant landrace PHB-0683 common bean (*Phaseolus vulgaris L.*), originated in Moncao (Portugal), was used [24]. Seeds were kindly provided by Prof. A. de Ron, from the Misión Biológica de Galicia’s seed collection (Pontevedra, Spain). Seeds were soaked in 96% ethanol for 30 s. Ethanol was discarded, and seeds were immersed in 5% sodium hypochlorite for 5 min. Then, seeds were repeatedly washed 6 times with sterile water and placed on moist paper on 120 mm Petri dishes for their imbibition at 26 °C and dim illumination during 72 h. After germination, three seedlings were sown on each pot (16 cm diameter, 18 cm height) filled with a mixture of vermiculite/perlite mixture (2/1 *w/w*) and inoculated with a fresh suspension of *Rhizobium leguminosarum* ISP 14, which had been cultured at 28 °C for less than 30 h. Inoculated plants were watered three times a week with nitrogen-free nutrient solution [58]. Plants were grown in a culture chamber with 300 μE.m^−2^. s^−1^ lighting for 16 h at 26 °C and 8 h of darkness at 20 °C and relative humidity of 70%, under well-irrigated conditions for four weeks, as previously described [24]. Four weeks after sowing, the plants were randomly separated into two sets, and the irrigation was withheld from one-half of the pots, and the second half was regularly watered with the standard nutrient solution to serve as controls.

Soil water capacity (SWC) was determined gravimetrically. Basically, pots filled with substrate were watered to excess, left to drain, and weighed to estimate the 100% SWC at sowing. The weighing was repeated during the drought treatments for both control and drought-stressed plants. SWC was maintained at 80%–90% for control plants during the whole experiment. Leaf relative water content (RWC) was estimated as RWC (%) ((Fw − Dw)/(Sw − Dw)) × 100. The water-saturated weight (Sw) of leaf samples was obtained by keeping leaf disks in distilled water at 4 °C for 12 h. Then, the samples were oven-dried at 70 °C to get a constant dry weight (Dw) [24].

### 4.2. Effect of Phosphate Addition on the Responses to Drought Stress

Plants under the condition of atmospheric nitrogen fixation were cultured and irrigated with the standard nitrogen-free nutrient solution containing 80 µM phosphate (normal P) until plants were 21 days old. Then, half of the plants were watered three times (in alternate days) with nutrient solution containing 200 µM phosphate (high P) for a week, whereas the second group was maintained under irrigation with the normal P solution. P-supplemented and control plants at 28 days old were randomly separated into two groups, one that received no further watering for 10 days (drought treatment), and the other that was irrigated with the regular nutrient solution (control). 

### 4.3. RNA-Seq Analysis

Plants cultured under standard nitrogen-fixing conditions for four weeks were randomly distributed into two groups, one of which was submitted to 10 days of water deprivation, whereas the group used as a control was regularly irrigated with the standard nitrogen-free nutrient solution. Three biological replicates, each consisting of the pooled 4th trifoliate leaves from 3–4 plants, from at least 3 independent control pots and three drought-treated pots, were used for RNA-seq analysis. Total RNA was isolated from the 6 samples by using Pure-link RNA isolation Kits (Thermo Fisher; Spain), according to the manufacturer’s instructions. RNA was quantified in a Nanodrop, and its quality was assessed in a 2100 bioanalyzer (Agilent). Poly A mRNA was isolated from 5 µg total RNA from each sample using Ambion Dynabeads™ mRNA Purification Kit (Thermo-Fisher) and used for cDNA libraries preparation using the Ion Total RNA-Seq Kit v2 for whole transcriptome libraries (Life Technologies Corporation, California, USA). cDNA libraries were loaded by an Ion Chef System, in three Ion 540 sequencing chips (each containing cDNA libraries from one control and one treated sample), and then further sequenced using an Ion S5 System (Thermo-Fisher Scientific). RNA-seq yielded approximately 33–37 million reads per library. The raw reads were analyzed for quality by FastQC [59] and processed to filter out poor quality sequences (Cutadapt version 1.9 (-m 100) and BBDuk version 35.43 (qtrim = rt trimq = 20)). The generated clean data were aligned to the reference genome for *P. vulgaris* L. v2.1 obtained from the Phytozome website (http://www.phytozome.net/) [31,60]. Genes were ranked according to normalized fragments per kilobase per million mapped reads (FPKM) to identify differentially expressed genes (DEGs). FPKM values were assigned to each gene by comparing the FPKM value under the drought treatment to that in the control condition. DegSeq2 R package was used to identify differentially expressed genes. Genes that were up- or downregulated at least 2-fold change (log2) with false discovery rate (FDR) adjusted *p*-value ≤ 0.05 were considered as DEGs [61,62].

### 4.4. GO Enrichment Analysis of DEGs

The bioinformatics analysis of DEGs was performed using Blast, Uniprot, and AgriGo v2.0 software (http://bioinfo.cau.edu.cn/agriGO/) [32] to determine the biological process, molecular functions, and cellular components enriched in the drought-treated samples.

### 4.5. Validation of DEGs by qRT-PCR Analysis

To validate RNA-Seq results, sixteen genes were selected from the list of DEGs and subjected to quantitative RT-PCR analysis. Gene-specific primer pairs (Supplementary Table S2) were designed by using Primer 3 + software and the qPCR default setting (http://www.bioinformatics.nl/cgi-bin/primer3plus/primer3plus.cgi). The total RNA was isolated using RNA-zol, according to the manufacturer’s instructions, and treated with RNase-free DNase I (New England Biolabs) at 37 °C for 10 min to eliminate polluting genomic DNA from samples. Next, first-strand cDNA synthesis was done from 2.5 µg of DNase-treated RNA using PrimeScript™ reverse transcriptase (TaKaRa) following the manufacturer’s instructions.

The expression analysis was carried out by qRT-PCR in an iCycler iQ System (Bio-Rad) using iQ SYBR-Green Supermix (Bio-Rad) and the specific primers for each gene (Supplementary Table S2). The program used consisted of an initial denaturation, together with a Taq polymerase activation, at 95 °C for 5 min followed by 40 cycles at 95 °C for 30 s, 60 °C for 30 s, and 72 °C for 30 s, and, finally, 80 cycles of 30 s at 60 °C. The relative expression of each gene in control and drought-stressed samples was calculated by the 2^−∆∆CT^ method [34], normalized to that of *Actin-2.* The quantification was performed using three independent biological replicates.

### 4.6. Promoter Analysis of the Phosphate-Related DEGs

The 5′ upstream regions (1.5 kb DNA sequence of each gene; Supplementary Table S3) were obtained from Phytozome database v12, and *cis*-elements scan was done using plant CARE software (http://bioinformatics.psb.ugent.be/webtools/plantcare/html/) [42].

### 4.7. Determination of Pi Concentration

Pi concentration was determined in leaf samples from control and drought-stressed plants cultured under standard P nutrition or which received a higher P concentration. The extraction protocol from leaf tissues was used as described in [63]. The Pi content was determined according to [64]. In brief, 50 mg leaf tissue was homogenized in 10 µL/mg of extraction buffer pH 8 (10 mM Tris-HCl, 1 mM EDTA, 100 mM NaCl, 1 mM β-mercaptoethanol, 1 mM PMSF). Then, samples were centrifuged at 11,000 g for 10 min, and 100 µL of the supernatants were mixed with 900 µL of 1% glacial acetic acid and incubated for 30 min at 42 °C. For Pi measurement, 300 µL of the extract was collected in a new tube to which 600 µL of molybdate solution (1 N H_2_SO_4_ and 0.42% NH_4_MoO_4_) and 100 µL of reducing solution (10% ascorbic acid) were added. Finally, the mixture was incubated at 45 °C for 20 min, and the absorbance at 820 nm was determined. The Pi concentration was obtained according to the calibration curve using known Pi concentrations.

### 4.8. Experimental Design and Statistical Analysis of The Data

A total of 18 plants were randomly divided between control plants and plants subjected to 10 days of drought for the RNA-seq drought experiment. Three independent biological replicates per condition, each from three independent plants, were used for the RNA-seq analysis. The whole experimental design from the other 18 plants was repeated to obtain the three biological replicates used in the qRT-PCR validation of RNA-seq DEGs.

In addition, another independent experiment was done in which 21 days old plants were separated into two groups, one of which was irrigated with nutrient solution enriched in P for one week. Then, the irrigation was withheld for 10 days for half of the 28 days old plants from the low and high P groups. Three replicas of each condition were used. Each replica consisted of a total of three plants per pot for each condition. Statistical analysis was done by Student’s *t*-test and ANOVA using GraphPad Prism 6 software package.

## Supplementary Materials

The following are available online at https://www.mdpi.com/2223-7747/9/4/445/s1, Table S1: Differentially expressed genes identified using expression profiling, Table S2: List of primers used in this study, Table S3: Cis-regulatory motives found in the selected DEGs. Figure S1. Quality Reports of RNA-seq analysis.

## References

[1] Broughton W.J., Hernández G., Blair M., Beebe S., Gepts P., Vanderleyden J. (2003). Beans (*Phaseolus* spp.)–model food legumes. Plant Soil.

[2] Beebe S.E., Rao I.M., Blair M.W., Acosta-Gallegos J.A. (2013). Phenotyping common beans for adaptation to drought. Front. Physiol..

[3] De Ron A.M., Papa R., Bitocchi E., González A.M., Debouck D.G., Brick M.A., Fourie D., Marsolais F., Beaver J., Geffroy V., De Ron A.M. (2019). Common bean. Grain Legumes.

[4] Graham P.H., Vance C.P. (2003). Legumes: Importance and constraints to greater use. Plant Physiol..

[5] Araújo S.S., Beebe S., Crespi M., Delbreil B., González E.M., Gruber V., Lejeune-Henaut I., Link W., Monteros M.J., Prats E. (2015). Abiotic Stress Responses in Legumes: Strategies Used to Cope with Environmental Challenges. Crit. Rev. Plant Sci..

[6] Serraj R., Vadez V., Denison R.F., Sinclair T.R. (1999). Involvement of ureides in nitrogen fixation inhibition in soybean. Plant Physiol..

[7] Sinclair T.R., Serraj R. (1995). Legume Nitrogen Fixation and Drought. Nature.

[8] Beebe S.E., Rao I.M., Cajiao V.C.H., Grajales M. (2008). Selection for drought resistance in common bean also improves yield in phosphorus limited and favourable environments. Crop Sci..

[9] McClean P.E., Burridge J., Beebe S., Rao I.M., Porch T.G. (2011). Crop improvement in the era of climate change: An integrated, multi-disciplinary approach for common bean (*Phaseolus vulgaris*). Funct. Plant Biol..

[10] Etienne P., Diquelou S., Prudent M., Salon C., Maillard A., Ourry A. (2018). Macro and Micronutrient Storage in Plants and Their Remobilization When Facing Scarcity: The Case of Drought. Agriculture.

[11] Rosales-Serna R., Kohashi-Shibata J., Acosta-Gallegos J.A. (2004). Biomass distribution, maturity acceleration and yield in drought-stressed common bean cultivars. Field Crop. Res..

[12] Frahm M.A., Rosas J.C., Mayek-Perez N., Lopez-Salinas E., Acosta-Gallegos J.A., Kelly J.D. (2004). Breeding beans for resistance to terminal drought in the lowland tropics. Euphytica.

[13] Terán H., Singh S.P. (2002). Comparison of sources and lines selected for drought resistance in common bean. Crop Sci..

[14] Muñoz-Perea C.G., Terán H., Allen R.G., Wright J.L., Westermann D.T., Singh S.P. (2006). Selection for drought resistance in dry bean landraces and cultivars. Crop Sci..

[15] Coleto I., Trenas A.T., Erban A., Kopka J., Pineda M., Alamillo J.M. (2016). Functional specialization of one copy of glutamine phosphoribosyl pyrophosphate amidotransferase in ureide production from symbiotically fixed nitrogen in *Phaseolus vulgaris*. Plants Cell Environ..

[16] Zrenner R., Stitt M., Sonnewald U., Boldt R. (2006). Pyrimidine and purine biosynthesis and degradation in plants. Annu. Rev. Plant Biol..

[17] Atkins C.A., Pate J.S., Ritchie A., Peoples M.B. (1982). Metabolism and translocation of allantoin in ureide-producing grain legumes. Plant Physiol..

[18] Díaz-Leal J.L., Gálvez-Valdivieso G., Fernández J., Pineda M., Alamillo J.M. (2012). Developmental effects on ureide levels are mediated by tissue-specific regulation of allantoinase in *Phaseolus vulgaris* L.. J. Exp. Bot..

[19] Alamillo J.M., Diaz-Leal J.L., Sanchez-Moran M.V., Pineda M. (2010). Molecular analysis of ureide accumulation under drought stress in *Phaseolus vulgaris* L.. Plant Cell Environ..

[20] Brychkova G., Alikulov Z., Fluhr R., Sagi M. (2008). A critical role for ureides in dark and senescence-induced purine remobilization is unmasked in the Atxdh1 Arabidopsis mutant. Plant J..

[21] Irani S., Todd C.D. (2016). Ureide metabolism under abiotic stress in *Arabidopsis thaliana*. J. Plant Physiol..

[22] Nourimand M., Todd C.D. (2017). Allantoin contributes to the stress response in cadmium-treated Arabidopsis roots. Plant Physiol. Biochem..

[23] King C.A., Purcell L.C. (2005). Inhibition of N_2_ fixation in soybean is associated with elevated ureides and amino acids. Plant Physiol..

[24] Coleto I., Pineda M., Rodiño A.P., de Ron A., Alamillo J.M. (2014). Comparison of inhibition of N2 fixation and ureide accumulation under water deficit in four common bean genotypes of contrasting drought tolerance. Ann. Bot..

[25] Cabeza R.A., Liese R., Lingner A., von Stieglitz I., Neumann J., Salinas-Riester G., Pommerenke C., Dittert K., Schulze J. (2014). RNA-seq transcriptome profiling reveals that *Medicago truncatula* nodules acclimate N_2_ fixation before emerging P deficiency reaches the nodules. J. Exp. Bot..

[26] Chen L.M., Zhou X.A., Li W.B., Chang W., Zhou R., Wang C., Chen S.L. (2013). Genome-wide transcriptional analysis of two soybean genotypes under dehydration and rehydration conditions. BMC Genomics.

[27] Jha U.C., Bohra A., Nayyar H. (2020). Advances in “omics” approaches to tackle drought stress in grain legumes. Plant Breed..

[28] Wu J., Wang L., Li L., Wang S. (2014). De novo assembly of the common bean transcriptome using short reads for the discovery of drought-responsive genes. PLoS ONE.

[29] Wu J., Wang L., Li L., Wang S. (2016). Comprehensive analysis and discovery of drought-related NAC transcription factors in common bean. BMC Plant Biol..

[30] Wu J., Chen J., Wang L., Wang S. (2017). Genome-Wide Investigation of WRKY Transcription Factors Involved in Terminal Drought Stress Response in Common Bean. Front. Plant Sci..

[31] Schmutz J., McClean P.E., Mamidi S., Wu G.A., Cannon S.B., Grimwood J., Jenkins J., Shu S., Song Q., Chavarro C. (2014). A reference genome for common bean and genome-wide analysis of dual domestications. Nat. Genet..

[32] Tian T., Liu Y., Yan H., You Q., Yi X., Du Z., Xu W., Su Z. (2017). agriGOv2.0: A GO analysis toolkit for the agricultural community, 2017 update. Nucleic Acids Res..

[33] Taji T., Ohsumi C., Iuchi S., Seki M., Kasuga M., Kobayashi M., Yamaguchi-Shinozaki K., Shinozaki K. (2002). Important roles of drought- and cold-inducible genes for galactinol synthase in stress tolerance in *Arabidopsis thaliana*. Plant J..

[34] Livak K.J., Schmittgen T.D. (2001). Analysis of relative gene expression data using real-time quantitative PCR and the 2(-Delta Delta C(T)) method. Methods.

[35] Denslow S.A., Rueschhoff E.E., Daub M.E. (2007). Regulation of the *Arabidopsis thaliana* vitamin B_6_ biosynthesis genes by abiotic stress. Plant Physiol. Biochem..

[36] Wild R., Gerasimaite R., Jung J.-Y., Truffault V., Pavlovic I., Schmidt A., Saiardi A., Jessen Y., Poirier H.J., Hothorn M. (2016). Control of eukaryotic phosphate homeostasis by inositol polyphosphate sensor domains. Science.

[37] Nakamura Y., Koizumi R., Shui G., Shimojima M., Wenk M., Ito T., Chrispeels M. (2009). Arabidopsis Lipins Mediate Eukaryotic Pathway of Lipid Metabolism and Cope Critically with Phosphate Starvation. Proc. Natl. Acad. Sci. USA.

[38] Cheng Y., Zhou W., El Sheery N.I., Peters C., Li M., Wang X., Huang J. (2011). Characterization of the Arabidopsis glycerophosphodiester phosphodiesterase (GDPD) family reveals a role of the plastid-localized AtGDPD1 in maintaining cellular phosphate homeostasis under phosphate starvation. Plant J..

[39] Schröder F., Lisso J., Lange P., Müssig C. (2009). The extracellular EXO protein mediates cell expansion in Arabidopsis leaves. BMC Plant Biol..

[40] Schröder F., Lisso J., Müssig C. (2011). EXORDIUM-LIKE1 promotes growth during low carbon availability in Arabidopsis. Plant Physiol..

[41] Wang W., Hu B., Li A., Chu C. (2019). NRT1.1s in plants: Functions beyond nitrate transport. J. Exp. Bot..

[42] Lescot M., Déhais P., Thijs G., Marchal K., Moreau Y., Van de Peer Y., Rouzé P., Rombauts S. (2002). PlantCARE, a database of plant cis-acting regulatory elements and a portal to tools for in silico analysis of promoter sequences. Nucleic Acids Res..

[43] Cutler S.R., Rodríguez P.L., Finkelstein R.R., Abrams S.R. (2010). Abscisic Acid: Emergence of a Core Signaling Network. Annu. Rev. Plant Biol..

[44] Baldoni E., Genga A., Cominelli E. (2015). Plant MYB Transcription Factors: Their Role in Drought Response Mechanisms. Int. J. Mol. Sci..

[45] Ding W., Fang W., Shi S., Zhao Y., Li X., Xiao K. (2016). Wheat WRKY type transcription factor gene TaWRKY1 is essential in mediating drought tolerance associated with an ABA-dependent pathway. Plant Mol. Biol. Rep..

[46] Joshi R., Wani S.H., Singh B., Bohra A., Dar Z.A., Lone A.A., Pareek A., Singla-Pareek S.L. (2016). Transcription Factors and Plants Response to Drought Stress: Current Understanding and Future Directions. Front. Plant Sci..

[47] Silva D.A.D., Tsai S.M., Chiorato A.F., da Silva Andrade S.C., Esteves J.A.F., Recchia G.H., Carbonell S.A.M. (2019). Analysis of the common bean (*Phaseolus vulgaris* L.) transcriptome regarding efficiency of phosphorus use. PLoS ONE.

[48] Chen J., Nolan T.M., Ye H., Zhang M., Tong H., Xin P., Chu J., Chu C., Li Z., Yin Y. (2017). Arabidopsis WRKY46, WRKY54, and WRKY70 Transcription Factors Are Involved in Brassinosteroid-Regulated Plant Growth and Drought Responses. Plant Cell.

[49] Besseau S., Li J., Palva E.T. (2012). WRKY54 andWRKY70 co-operate as negative regulators of leaf senescence in *Arabidopsis thaliana*. J. Exp. Bot..

[50] Ezquer I., Salameh I., Colombo L., Kalaitzis P. (2020). Plant Cell Walls Tackling Climate Change: Insights into Plant Cell Wall Remodeling, Its Regulation, and Biotechnological Strategies to Improve Crop Adaptations and Photosynthesis in Response to Global Warming. Plants.

[51] Le Gall H., Philippe F., Domon J.M., Gillet F., Pelloux J., Rayon C. (2015). Cell Wall Metabolism in Response to Abiotic Stress. Plants.

[52] Sa T.M., Israel D.W. (1991). Energy status and functioning of phosphorus-deficient soybean nodules. Plant Physiol..

[53] Schultze J., Temple G., Temple S., Beschow H., Vance C.P. (2006). White lupin nitrogen fixation under phosphorus deficiency. Ann. Bot..

[54] Hernández G., Ramírez M., Valdés-López O., Tesfaye M., Graham M.A., Czechowski T., Schlereth A., Wandrey M., Erban A., Cheung F. (2007). Phosphorus Stress in Common Bean: Root Transcript and Metabolic Responses. Plant Physiol..

[55] Raschke M., Boycheva S., Crèvecoeur M., Nunes-Nesi A., Witt S., Fernie A.R., Amrhein N., Fitzpatrick T.B. (2011). Enhanced levels of vitamin B_6_ increase aerial organ size and positively affect stress tolerance in Arabidopsis. Plant J..

[56] Li D., Zhang T., Wang M., Liu Y., Brestic M., Chen T.H.H., Yang X. (2019). Genetic Engineering of the Biosynthesis of Glycine Betaine Modulates Phosphate Homeostasis by Regulating Phosphate Acquisition in Tomato. Front. Plant Sci..

[57] Bargaz A., Nassar R.M.A., Rady M.M., Gaballah M., Thompson S.M., Brestic M., Schmidhalter U., Abdelhamid M.T. (2016). Recombinant Inbred Lines Contrasting in Their P-Efficiency.Improved Salinity Tolerance by Phosphorus Fertilizer in Two *Phaseolus vulgaris* Recombinant Inbred Lines Contrasting in Their P-Efficiency. J. Agron. Crop Sci..

[58] Rigaud J., Puppo A. (1975). Indole-3-acetic Acid Catabolism by Soybean Bacteroids. J. Gen. Microbiol..

[59] Andrews S. (2010). Babraham Bioinformatics-FastQC a Quality Control Tool for High Throughput Sequence Data. https://www.bioinformatics.babraham.ac.uk/projects/fastqc/.

[60] Ghosh S., Chan C.K.K., Edwards D. (2016). Analysis of RNA-Seq Data Using TopHat and Cufflinks. Plant Bioinformatics.

[61] Love M.I., Huber W., Anders S. (2014). Moderated estimation of fold change and dispersion for RNA-seq data with DESeq2. Genome Biol..

[62] Love R., Shirley N., Bleackley M., Dolan S., Shafee T. (2017). Transcriptomics technologies. Plos Comput. Biol..

[63] Chiou T.J., Aung K., Lin S.I., Wu C.C., Chiang S.F., Su C.L. (2006). Regulation of Phosphate Homeostasis by MicroRNA in Arabidopsis. Plant Cell.

[64] Ames B.N. (1966). Assay of inorganic phosphate, total phosphate and phosphatases. Methods Enzym..

