# Supplementary material for: Transcriptomic Response to Water Deficit Reveals a Crucial Role of Phosphate Acquisition in a Drought-Tolerant Common Bean Landrace
Source: Plants (Basel). 2020 Apr 2;9(4):445. doi: 10.3390/plants9040445 (PMC7238123; doi:10.3390/plants9040445)
Supplement: Supplementary file 1 [file plants-09-00445-s001.zip › Table S2. List of Primers.docx]

Table S2. List of primers used in the qRT-PCR analysis

| **ID gene** | **Gene name** | **Primer (5` 🡪 3´)** |
| --- | --- | --- |
| *Phvul.001G021200* | *PvPP2C-12* | FW-GGCTTTAGTTGCTGGCTTTG |
|  |  | Rev-TGCCACTGTTACAACCCAAC |
| Phvul.002G122200 | *ABA8H* | FW-AAGTTCGTGCTCCACAAAGC |
|  |  | Rev-AAATTGCTTGCGTCCCCAAC |
| *Phvul.008G185700* | *WRKY70* | FW-AACGTCTCCACATTGCAGTG |
|  |  | Rev-TCAGTGGTGCAAGAGACTATGG |
| *Phvul.003G028000* | *MYB* | FW-GTTCACAAGCAGCCCCTTTG |
|  |  | Rev-TGATGAGGTAGCGACTGCAC |
| Phvul.001G091000 | *PvGDP.CDPK* | Fw-TTCTCGGAGGATAATGGTGCTG |
|  |  | Rev-TTTCCCTCATTCCCATGCTCT |
| Phvul.002G061900 | *PvNRT1* | Fw-TTGAAATTCCCGCAGCATCG |
|  |  | Rev-TGCCACCGGAACAATGAAAC |
| Phvul.003G164900 | *PvSPX3* | Fw-TTTCCACGGAGAGATGGTCTTG |
|  |  | Rev-TTCTGAATGAAGGGCAAGCG |
| Phvul.009G197000 | *PvSPX9* | Fw-AGCCAACCAGCACATTGATG |
|  |  | Rev-CGGTGGCAATGAAAACATGC |
| Phvul.010G140900 | *PvPhospho9* | Fw-CAACTGGGTCGTCGATGAATTG |
|  |  | Rev-TGTCCATGAGAGTGTTCCAAGG |
| Phvul.010G141200 | *PvPhospho12* | Fw-CGTGGATGAATTGGGTTTCACC |
|  |  | Rev-TGTGTCCATGAGAGTGTTCCAG |
| Phvul.010G140800 | *PvPhospho8* | Fw-AGATGGCAGTGGAGACTATTGC |
|  |  | Rev-TATCAAGTCCCACACCGGAAAG |
| Phvul.001G021400 | *PvLPIN* | Fw-TTGTGTCGAGGTCTTTGAGAGG |
|  |  | Rev-CCACTTCAGCTCCAACAAGTTG |
| Phvul.008G038300 | *PvPHO1-2* | Fw-AGGCCTTGTCATTGTTCTGC |
|  |  | Rev-AGAGGAGCACAAACGCAATG |
| Phvul.008G176000 | *PvPhi 1* | Fw-TCGGCAAATCACTCACCAAC |
|  |  | Rev-AACCACGTTGATGGCGTTTC |
| Phvul.011G004400 | *PvPhi 1-L (EXD7)* | Fw-AAGAGGGGCATGTGCAAATG |
|  |  | Rev-TCGAATCGCTTGGAGGAAGAG |
| Phvul.009G032100 | *PvPhi 1-L (EXD9)* | Fw-ACGGTCCTTTGCTTTATGGC |
|  |  | Rev-TTTTGGGAGGGTTTGAAGCG |
| KF033666.1 | *PvACT-2* | Fw-GGAGAAGATTTGGCATCACACGTT |
|  |  | Rev-GTTGGCCTTGGGATTGAGTGGT |
